# The patient at the centre: evidence from 17 European integrated care programmes for persons with complex needs

**DOI:** 10.1186/s12913-020-05917-9

**Published:** 2020-11-30

**Authors:** Thomas Czypionka, Markus Kraus, Miriam Reiss, Erik Baltaxe, Josep Roca, Sabine Ruths, Jonathan Stokes, Verena Struckmann, Romana Tandara Haček, Antal Zemplényi, Maaike Hoedemakers, Maureen Rutten-van Mölken

**Affiliations:** 1 Institute for Advanced Studies, Josefstädter Straße 39, 1080 Vienna, Austria; 2 London School of Economics and Political Science, Houghton Street, London, WC2A 2AE UK; 3 Hospital Clinic de Barcelona, Institut d’Investigacions Biomèdiques August Pi i Sunyer (IDIBAPS), Universitat de Barcelona, Villarroel 170, Barcelona, 08036, Catalonia, Spain; 4 University of Bergen, Postboks 7804, 5020 Bergen, Norway; 5 University of Manchester, 7th Floor, Williamson Building, Oxford Road, Manchester, M13 9P UK; 6 Berlin University of Technology, Strasse des 17. Juni 135 (H80), 10623 Berlin, Germany; 7 Ministry of Health, Ksaver 200a, 10000 Zagreb, Croatia; 8 Syreon Research Institute, Mexikoi str. 65/A, 1142 Budapest, Hungary; 9 Erasmus University Rotterdam, P.O.Box 1738, 3000 DR Rotterdam, The Netherlands

**Keywords:** Integrated care, Complex needs, Thick description, Person-centred care, Delivery of care

## Abstract

**Background:**

As the prevalence of multi-morbidity increases in ageing societies, health and social care systems face the challenge of providing adequate care to persons with complex needs. Approaches that integrate care across sectors and disciplines have been increasingly developed and implemented in European countries in order to tackle this challenge. The aim of the article is to identify success factors and crucial elements in the process of integrated care delivery for persons with complex needs as seen from the practical perspective of the involved stakeholders (patients, professionals, informal caregivers, managers, initiators, payers).

**Methods:**

Seventeen integrated care programmes for persons with complex needs in 8 European countries were investigated using a qualitative approach, namely thick description, based on semi-structured interviews and document analysis. In total, 233 face-to-face interviews were conducted with stakeholders of the programmes between March and September 2016. Meta-analysis of the individual thick description reports was performed with a focus on the process of care delivery.

**Results:**

Four categories that emerged from the overarching analysis are discussed in the article: (1) a holistic view of the patient, considering both mental health and the social situation in addition to physical health, (2) continuity of care in the form of single contact points, alignment of services and good relationships between patients and professionals, (3) relationships between professionals built on trust and facilitated by continuous communication, and (4) patient involvement in goal-setting and decision-making, allowing patients to adapt to reorganised service delivery.

**Conclusions:**

We were able to identify several key aspects for a well-functioning integrated care process for complex patients and how these are put into actual practice. The article sets itself apart from the existing literature by specifically focussing on the growing share of the population with complex care needs and by providing an analysis of actual processes and interpersonal relationships that shape integrated care in practice, incorporating evidence from a variety of programmes in several countries.

**Supplementary Information:**

The online version contains supplementary material available at 10.1186/s12913-020-05917-9.

## Background

European societies, as many other societies worldwide, face the challenge of an increasing number of persons with multi-morbidity, which is commonly defined as two or more chronic conditions occurring in one person at the same time [1–3]. Multi-morbidity is associated with lower quality of life [4–6] and poor experience of care [7, 8] for the affected persons. Furthermore, multi-morbidity imposes high costs on healthcare systems [9–11]. Data on prevalence vary greatly depending on definitions and methodologies [3], but it is commonly estimated that more than half of the population aged 65 or older is affected by multi-morbidity in Western societies [1, 2, 12–15].

The care of persons with multi-morbidity places high demands on health and social care systems as these individuals often require services from multiple providers in both systems. This part of the population has therefore been suggested to particularly benefit from innovative care models that aim to integrate care across sectors and disciplines [16–19]. In recent years, such innovative integrated care models for persons with multi-morbidity have been increasingly developed and implemented in European countries [20, 21].

The topic of integrated care for multi-morbidity is currently being tackled by the SELFIE project (Sustainable intEgrated chronic care modeLs for multi-morbidity: delivery, FInancing, and performance; www.selfie2020.eu), a research project funded by the Horizon2020 programme of the European Commission. Within the SELFIE project, integrated care is defined as structured efforts to provide coordinated, pro-active, person-centred, multidisciplinary care by two or more well-communicating and collaborating care providers either within or across sectors [22]. In the course of the project, 17 successful integrated chronic care programmes in the eight partner countries of the SELFIE consortium (Austria, Croatia, Germany, Hungary, the Netherlands, Norway, Spain, UK) were identified in order to be investigated and evaluated (see Table 1) [23]. As these programmes target a broad spectrum of complex health as well as social needs, we will predominantly use the term “complex needs” rather than “multi-morbidity” in the following.

**Table 1:** Basic information on the 17 selected integrated care programmes for persons with complex needs

	Programme name	Location	Programme type	Target group	Aim
*P01*	*Health Network Tennengau*	Tennengau region, Salzburg, Austria	Bottom-up network of social and health service providers and voluntary organisations	Entire population of the Tennengau region, but particular focus on elderly persons in need of social care	Improving coordination of care across sectors and providers; improving patient experience
*P02*	*Sociomedical Centre Liebenau*	Liebenau and Jakomini districts in the city of Graz, Styria, Austria	Multi-disciplinary group practice collaborating with association for practical social medicine	Persons with complex needs in multiple life domains (e.g. physical/mental health problems, social problems)	Providing holistic health and psychosocial care to vulnerable groups according to an emancipatory approach
*P03*	*GeroS*	Croatia (covers several counties)	Information system for health and social care records	All insurees aged 65 and over, in particular geriatric patients	Centralising of health and social care data; monitoring and evaluating health needs and functional abilities of the elderly population
*P04*	*Palliative Care System*	Croatia (covers several counties)	Coordination programme for palliative care	Persons in need of palliative care	Improving quality and adequacy of palliative care; implementing systematic care approach on a national level
*P05*	*Casaplus*	Germany (covers entire country)	Case management programme contracted by sickness funds	Persons aged 55 and over with multiple chronic conditions and at high risk for hospitalisation	Reducing avoidable hospitalisations through preventive case management and enhanced self-management skills
*P06*	*Gesundes Kinzigtal*	Kinzigtal region, Baden-Württemberg, Germany	Population-based integrated care initiative	Entire population of the Kinzigtal region	Improving health of the population and patient experience; reducing per-capita costs of care
*P07*	*OnkoNetwork*	Somogy county, Hungary	Coordination programme in an oncology centre	Persons with (suspected) diagnosis of a solid tumor	Improving clinical outcomes for oncology patients via timely access to care and patient pathway management tools
*P08*	*Palliative Care Consult Service*	Baranya county, Hungary	Consultation programme for palliative care	Persons in need of palliative care	Providing high-quality palliative care to patients as well as support to families and professionals
*P09*	*Proactive Primary Care Approach for Frail Elderly (U-PROFIT)*	Utrecht and North-West Veluwe regions, Netherlands	Nurse-led elderly care intervention	Frail elderly persons aged 60 years and over living at home	Transitioning from reactive to proactive elderly care; preserving daily functioning; improving quality of care; reducing costs of care
*P10*	*Care Chain Frail Elderly*	South-East Brabant region, Netherlands	Multi-disciplinary care chain	Elderly persons with complex care needs living at home	Improving functional ability, health status and well-being; preventing/postponing nursing home admission
*P11*	*Better Together in Amsterdam North (BSiN)*	Amsterdam North district in the city of Amsterdam, Netherlands	Alliance of organisations from healthcare, social care, welfare, social security and youth care	Persons with complex needs in multiple life domains (e.g. physical/mental health problems, social problems)	Improving health and self-sufficiency of target population; improving quality of care; reducing costs of care
*P12*	*Medically Assisted Rehabilitation Bergen*	City of Bergen, Norway	Multi-disciplinary specialised treatment programme for opioid addiction	Persons with opioid addiction	Providing low-threshold integrated care beyond addiction treatment; improving quality-adjusted life expectancy
*P13*	*Learning Networks*	Municipalities across Norway	Multi-disciplinary integrated care teams in municipalities	Elderly persons using home nursing services or with short-term stays in nursing homes	Developing coordinated and safe patient pathways and health promotion services; improving functional ability
*P14*	*Badalona Serveis Assistencials*	Badalona region, Spain	Integrated care organisation of health and social service providers	Frail elderly persons with complex care needs	Promoting independent living by offering support to prevent hospitalisation and nursing home admission
*P15*	*Área Integral de Salut, Barcelona Esquerra (Ais-Be)*	Barcelona-Esquerra, city of Barcelona, Catalonia, Spain	Programme for community-based collaborative care by a university hospital	Persons with complex care needs	Bridging between hospital-based specialised care and community-based services
*P16*	*Salford Integrated Care / Salford Together*	City of Salford, Greater Manchester, United Kingdom	Community-based integrated chronic care programme	Adults with chronic conditions	Improving coordination of care; supporting patients in self-management; reducing hospitalisations and nursing home admissions
*P17*	*South Somerset Symphony*	South Somerset district, United Kingdom	Health coaching programme in hospital-based complex care hubs and GP practices	Persons with 3 or more chronic conditions	Supporting patients in self-management and thereby empowering them; improving coordination of care

One of the aims of the SELFIE project was to go beyond theory and quantitative evaluations, and use experiences of defined groups of stakeholders to gain a deeper insight into the inner workings of the programmes – in particular, why they are successful in improving care for persons with complex needs. Such insights can serve as guidance in the future implementation of integrated care models for persons with complex needs. The aim of the current article is therefore to sum up our findings on success factors as well as crucial elements of integrated care programmes for persons with complex needs as seen from the practical perspective of the involved stakeholders. A particular focus is placed on the process of care delivery and the implicit social structures associated with it. The methodological approach chosen for this purpose, namely “thick description”, draws on the personal views of the persons involved in the care process as well as objective information. The material is condensed into emerging common themes and the way they are addressed in the care process.

## Methods

### Thick description of individual programmes

The methodological approach of thick description was chosen to investigate the functioning of integrated care programmes for persons with complex needs going beyond descriptive and quantitative information. In the 1940s, the idea of thick description was introduced by the philosopher Gilbert Ryle [24], and in the 1970s, it was further developed and established as a qualitative approach to investigate implicit social practices in their specific contexts by the anthropologist Clifford Geertz [25]. In recent decades, thick description has been widely used in a variety of research fields, including research of care practices (e.g. [26–28]).

**Fig. 1:**
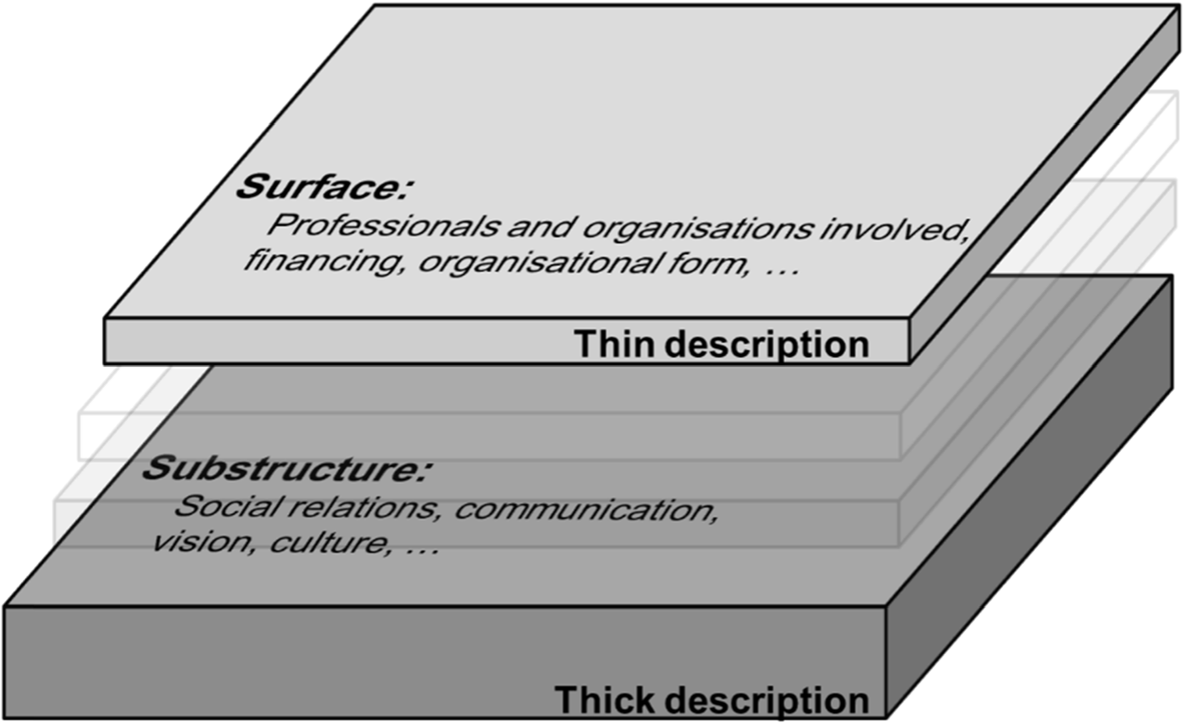
Visualisation of thick description of integrated care programmes for persons with complex needs

Thick description aims to investigate patterns of cultural and social relationships while taking into account the specific context of the studied case. This involves the social actions and the circumstances under which they take place, such as thoughts, feelings and the web of relationships between the participants. Thick description does not merely provide superficial information on the studied case (e.g. services provided, professionals involved, target group, financing, organisational form), but reaches further into underlying social patterns and substructures (e.g. relationships, social roles), in contrast to its logical opposite, thin description [29–32]. The method was chosen for this specific research context because it allows for an open and inductive approach to investigating care processes. Figure 1 shows a visualisation of this approach in the context of our analysis. In practice, thick description can be based on various kinds of materials, e.g. participant observation, qualitative interviews, written documents or video/audio recordings.

Each of the 17 integrated care programmes for persons with complex needs was individually investigated by means of thick description by the researchers of the SELFIE team from the respective country. In order to gather sufficient data for an analysis of this kind, document analysis and, more importantly, semi-structured interviews were conducted. The Austrian SELFIE team assumed the conceptualising and coordinating role in this research process, as it was leading the corresponding work package within the SELFIE project due to their comprehensive expertise in the field. The Austrian SELFIE team prepared a written guideline for both document analysis and interviews and held a workshop on the methodological approach (including analysing documents, conducting and analysing interviews, synthesising evidence into thick description reports) for all participating experts in order to ensure a uniform approach and homogeneity of the analysis in all partner countries. Furthermore, the Austrian team was in constant exchange with the researchers in all partner countries during the entire process of data collection and analysis.

Thick description of the individual programmes was conducted by the SELFIE team members from each respective country. Each of the researchers held a degree from social sciences and/or medicine at the level of PhD candidate or higher. In each country, the individual research team consisted of both women and men. Overall, exactly 50% of the research team were female.

#### Document analysis

The document analysis aimed to describe the general organisational structure of the programme as well as the formal relations of the involved stakeholders, representing the “thin description”. In particular, it intended to provide the following information on the programme: aim, starting date, geographical scope, target group, services provided, number of persons treated, organisational form, involved partner organisations, involved disciplines and professions. For this purpose, available literature (scientific articles, academic theses, research reports, grey literature) and other documents (official documents, contracts, brochures, website of the programme) were screened for relevant information by researchers of the SELFIE team from the respective partner countries.

#### Semi-structured interviews

The semi-structured interviews aimed to gain insights in what actually constitutes the programme below its surface when put into practice, in particular with regard to the care process. Interviews were conducted with different predefined stakeholder types involved in the programme, namely patients, professionals (e.g. medical and social staff), informal caregivers, manager(s) of the programme, initiator(s) (i.e. persons who were involved in setting up the programme), representatives of sponsor/payer organisations and other stakeholders.

##### Interview guides for stakeholder types

Thematic focus areas for the interviews were defined inductively for each stakeholder type. Based on this, an individual interview guide was developed for each stakeholder type in order to account for different backgrounds and relevant themes of the individual stakeholder types (see Supplementary materials). This approach allows for gaining insights into the programme from different perspectives. The included questions concerned, for example, the stakeholders’ perceptions of the care process, their roles and relationships within the programme, their specific problems and applied solutions, and their personal views on the programme. The interview guides were pilot tested by the Austrian SELFIE team with several stakeholders of the Health Network Tennengau. Researchers in the partner countries were free to adapt the interview guides according to specifics of their cases (e.g. cultural particularities) in close consultation with the Austrian team.

##### Selection of interview partners

On the basis of the document analysis, a purposive sample of interviewees was defined for each individual programme, as the importance of types and compositions of stakeholders varied across the programmes. The samples were defined by the researchers in the respective partner countries and subsequently discussed with the Austrian SELFIE team. Potential interviewees had no prior relationship to the researchers and were approached personally, by email or by telephone. A minimum number of 10 persons had to be interviewed per programme (at least one per stakeholder group). Researchers were free to invite additional interviewees in case they regarded it necessary to obtain more information. In general, interviewees in the different stakeholder groups showed high willingness to participate. Only in very few cases, potential interviewees dropped out, e.g. two persons in Germany (one due to pregnancy, one due to illness). In total, 233 interviews were conducted (28 patients, 100 professionals, 19 informal caregivers, 33 mangers, 20 initiators, 22 representatives of sponsor/payer organisations and 11 others stakeholders). The average number per programme was 13.7.

##### Conducting interviews

Interviews were conducted face-to-face between March and September 2016 by researchers of the SELFIE team from the respective partner countries. All interviewees gave informed consent at the beginning of the interview. Prior to giving their informed consent, each interviewee was informed about the SELFIE project as well as about the background of the respective interviewer (e.g. educational/occupational background, research interests). The interviews lasted approx. Thirty to ninety minutes and were digitally audio-recorded. Some researchers took field notes in addition to the audio recordings. For professionals, managers, initiators and payers, the interviews usually took place at the workplace of the interview. For patients and informal caregivers, the interviews usually took place at the home of the interviewee or at a programme-related facility. Generally, there was no one present during the interviews besides researchers and interviewees. Instructions were clear that interviewees could terminate or pause the interview at any point. No participant payment was made to the interviewees.

##### Analysis

All interviews were transcribed verbatim from the audio file either by the interviewer or an independent research transcriber. The resulting transcripts were analysed using Mayring’s qualitative content analysis [33]. The analysis was guided by a broad category system based on the six components of the SELFIE conceptual framework of integrated care for multi-morbidity (service delivery, leadership and governance, workforce, financing, technologies and medical products, and information and research) [22]. Sub-categories within these broad components, which were relevant to the specific cases, were identified inductively. The units of analysis, i.e. verbal sequences from the interviews, were coded according to the categories and sub-categories by researchers of the SELFIE team from the respective country (2 per team). No specific software was used for managing the data. A thick description report was produced for each of the 17 programmes. The interview quotations selected to be included in the reports were edited into readable forms and translated into English. The reports (to be found on www.selfie2020.eu/publications) were structured according to the six components of the conceptual framework and also contain further information on the methodological approach.

### Overarching analysis

The thick description reports of all 17 integrated care programmes were subjected to a meta-analysis [34, 35] focussing on the process of care delivery. The analysis was led by the research question “Which aspects of the care process are crucial to integrated care for persons with complex needs and in which way do these aspects contribute to the success of such initiatives?”. Two researchers from the Austrian SELFIE team conducted independent analyses of the themes addressed in the reports in order to inductively identify categories associated with a well-functioning care process on the practical level. Coding and interpretation of results were discussed to explore differences in interpretation of narratives, improve consistency of coding, and reduce subjective influences. The categories were checked back against the thick description reports to ensure consistency and validity.

## Results

While the 17 programmes differ from each other in various ways (e.g. target group, setting, cultural background, health system context), the overarching analysis focuses on success factors and key elements that are common across the programmes and contribute to a well-functioning care process. We grouped the main themes that emerged from the analysis into the following four categories: holistic view of the patient, continuity of care, relationships between professionals, and patient involvement.

### Holistic view of the patient

There is increasing consensus that integrated care of persons with complex needs cannot exclusively address physical health problems, but needs to adopt a holistic view of the person [36–40]. This is based on the recognition that physical health, mental health and the social situation are interconnected and should therefore not be dealt with independently. This focus on a holistic care approach has likewise emerged in our analysis. The two sub-themes we identified in this context are (1) consideration of mental health and (2) consideration of the social situation of patients.

#### Consideration of mental health

In accordance with this holistic view, most programmes take into account mental health in the care process and many provide corresponding services as integrated part of the programme (e.g. psychotherapy, counselling). Both a care coordinator and a programme initiator from South Somerset Symphony [UK] acknowledge that the presence of mental health problems increases complexity and poses additional challenges in the provision of care:

*“I think anxiety and depression are huge and I certainly didn’t realise how much that impacts on a person’s health and wellbeing and, you know, some people can have three, four [physical] long term conditions and can manage quite well, somebody that could have anxiety and depression could have one [physical] long term condition and it’s, you know, they don’t manage at all.” (P17_IP09)*

*“[...] issues around loneliness, social isolation, anxiety, mental health issues, such a widespread anxiety and depression issues, confidence issues, a whole range of things that can’t be changed overnight. That takes time to work with individuals and their families and their carers… I think the most challenging individuals are those with severe and enduring mental health issues.” (P17_IP03)*

#### Consideration of social situation of patients

Apart from considering physical and mental health problems, most of the investigated programmes also put emphasis on the social situation of their patients. While some programmes at least take into account the patient’s social context when planning the care process, others even strive to design care so as to actively target social problems.

The philosophy of the Sociomedical Centre Liebenau [AT], for example, is guided by a “social medicine” approach. Based on a firm belief in the significance of social determinants of health, the programme aims at improving patients’ social situation in addition to providing healthcare in a narrower sense. To this purpose, it employs social workers, provides various counselling services and runs a community centre. A physician points out that patients often prioritise social aspects over their physical health:

*“[...] if someone doesn’t know how he is going to finance his everyday needs, then coping, for instance, with their diabetes or their multiple illnesses is probably the least of their worries, because they’ll say: ‘Okay, that’s an organic illness that I have, but I don’t know if I can keep the apartment or I don’t know if the youth welfare office is going to take my children away or something.’ As a doctor, I then have the responsibility to also help resolve these problems, because only then will the medicine prescribed work.” (P02_IP04)*

Medically Assisted Rehabilitation Bergen [NO], which specifically targets drug users, also provides social support to its patients. As a health professional involved in the programme highlights, it is necessary to secure the patient’s basic needs before starting the actual treatment of health problems:

*“My way of thinking has been that you must attend to the basics first, before you move on. For many, the opioid addiction or the search for heroin will be the one thing that overshadows everything else, it takes control over all other needs, so if you got that right, then you need to secure housing, and attend to economic problems and then you can start to dig into [mental problems].” (P12_IP03)*

In order to fully capture the social situation of persons with complex needs, the importance of personal contact to patients in their own environment during the assessment was stressed by several interview partners. This allows for a better understanding of the patient’s situation and the scale of his/her complex needs, as a nurse from Badalona Serveis Assistencials [ES] highlighted:

*“We think the first visit is very important and it must be done at home, because then you can see which is the social situation, the environment, if the patient is ready to follow our instructions, if he takes the medication … This is an important issue, because when you are at the consultation, they say ‘Yes, I take this, I take that’, but when you go to their homes and open the medicine cabinet, it’s a mess and you see that there are many things they don’t take, or that they don’t do it well.” (P14_IP14)*

### Continuity of care

The concept of continuity of care involves a continuous caring relationship with an identified health care professional and seamless and timely service provision across multiple providers [41]. It is a central aspect of quality of care and especially important when providing care to persons with complex needs [36, 42, 43]. Continuity of care was identified as a central aspect to the success of the investigated programmes. In particular, three sub-themes emerged, namely (1) the existence of a single contact point for patients, (2) the alignment of the services offered by a programme and (3) the relationship between patients and non-physician professionals.

#### Existence of single contact point for patients

Many of the investigated programmes involve certain professionals acting as a single contact point for patients. Some examples are case managers in Casaplus, elderly care nurses in U-PROFIT, special advisors in Medically Assisted Rehabilitation Bergen or case management nurses in Badalona Serveis Assistencials. Depending on the primary focus of the programme these persons have different professional backgrounds (e.g. nurses, social workers). Persons with complex needs, their informal caregivers but also professionals and programme managers appear to highly value the existence of such a single contact point ensuring a targeted navigation through the health and social care system, as the latter is often a challenging task for these persons.

In South Somerset Symphony [UK], patients are assigned a care coordinator who acts as such a single contact point and manages transition from multiple care pathways to a single coordinated and integrated pathway. Patients perceive a benefit to their care from this service:

*“It doesn’t matter what is wrong with me, I can discuss it with them. If I need a doctor’s appointment, they can make one at the surgery for me and they can … if it's something to do with, say, the diabetes and they think I need a review, they will arrange all of that for me. So it is, as they have said, one body of people I can go to that has access to everything I need.” (P17_IP04)*

A similar role is played by the case management nurse in Ais-Be [ES], who coordinates the different specialists involved in a patient’s treatment. Furthermore, when the patient is stable, the case management nurse is in charge of monitoring and follow-up. In the following, a member of the innovation directorate emphasises the significance of the case management nurse:

*“This is the role of the case manager, to put order in the confusion generated by different doctors that are seeing only one part of the patient. This fragmentation is the one solved by the case manager [ …] I would say that the strength of the case manager is giving continuity to the patient, monitoring the patient with low intensity but frequently.” (P15_IP05)*

#### Alignment of services offered

Addressing health issues of patients with multiple chronic conditions requires multiple different professionals to deliver appropriate care for both health and social problems in a coordinated way. This can be specifically challenging for persons with complex needs. At the centre of integrated care programmes for persons with complex needs, there is often a multi-disciplinary team covering a potentially broad spectrum of professions from the health and social care fields [44, 45]. This team-based approach is appreciated by both the professionals themselves and patients.

The team of the Sociomedical Centre Liebenau [AT], for example, includes health professionals, a psychotherapist, social workers and a legal advisor – all under one roof. This enables the Sociomedical Centre Liebenau to act as a “one-stop shop” and provides low-threshold access to a variety of services. A social worker, for example, stresses that her presence at the centre encourages patients to use her services who would otherwise be reluctant to do so:

*“It’s quite possible for people to go to their GP, and for him to ask: ‘Have you talked to a social worker about that?’ And for them to say: ‘No, I haven’t, but I’ll think about it.’ But then there’s usually a psychological barrier. But if the doctor says: ‘Wait a moment, we have a social worker here, you can meet them right away.’ When the people then see me, and I start talking to them and building a relationship at once, it’s easier for many people, and they can come to one and the same place for different problems, don’t need to go to yet another place.” (P02_IP02)*

#### Relationship between patients and non-physician professionals

In several programmes, addressing and making use of the distinctive nature of the relationship between patients and non-physician professionals has emerged as a central aspect. Non-physician professionals play a key role in many of the programmes; as a consequence, patients and/or informal caregivers develop special relationships to them.

In Casaplus [DE], a relationship of mutual trust and learning has developed over time between many patients and their case managers as a consequence of regular contact. The case management approach is aimed at complementing the physician’s treatment and is viewed by professionals and patients as a valuable addition. A patient, for example, appreciates receiving more extensive advice on handling her diseases:

*“Well, I’m not a physician, but the case managers there have a lot more knowledge and influence, thus they explain diseases and their potential consequences and other important things to me.” (P05_IP08)*

In U-PROFIT [NL], an elderly care nurse takes on the central role in the care process. His/her role is perceived as important in frail elderly care in general, but especially in complex cases that go beyond the medical domain. Several aspects in this context were highlighted in the interviews, such as the patients’ special relationship to the elderly care nurse in contrast to their relationship to the physician. In particular, patients are more willing to share concerns with the elderly care nurse, as a physician of the programme acknowledges:

*“[…] patients are open in a really different way towards the nurses than towards us [GPs]. Often much more is said, they dare to say much more, because then you don’t bother the GP even though you [the GP] think they can really say more, they just don’t.” (P09_IP04)*

A feature that patients particularly value with regard to non-physician professionals, seems to be the time these professionals spend with them, as a patient in BSiN [NL] emphasises:

*“Just making the time […] that the time is just there you know, that is nice. That you don’t feel the pressure ‘oh now we have to do this quickly’ because she has to leave in three minutes for example.” (P11_IP05)*

### Relationships between professionals

While the quality of relationships between professionals always plays a role in the delivery of care, it is of particular importance when various disciplines are involved and cases are complex [46–48]. Hence, this aspect has been raised in the context of various programmes as a prerequisite for a well-functioning care process. We identified two sub-themes in this context: (1) building trust between professionals, and (2) communication between professionals.

#### Building trust between professionals

Several stakeholders of the investigated programmes stressed that good collaboration can only be achieved if all involved partners form trusting relationships with each other. This is particularly relevant when professionals are not used to collaborating closely because standard care settings do not require them to do so. However, it was also acknowledged that building such relationships requires effort, time and a team culture that allows for open-minded discussion.

In the following, a care manager and initiator of the Health Network Tennengau [AT] states that he appreciates the openness in communication between the professionals involved in the network, which also allow for expression of criticism:

*“I think a certain culture has developed over the years in the Tennengau region. Nowadays, there are no borders between the different participants. If I contact someone, that contact is basically friendly and positive from the start, even if I were perhaps on occasion to voice criticism. […] I’ve heard that in other areas that can often cause tensions, that people are in competition with each other. […] We support and encourage each other and that’s what I find good and is what, I think, has established itself over the course of time.” (P01_IP05)*

Similarly, Salford Integrated Care [UK] has a long history of collaboration, which dates back to before the programme was implemented in its current form. According to the programme manager, the trust built over time was a central prerequisite for successful programme development:

*“I think our history of partnership working is the most important issue, and the relationships and the trust and the respect that’s been built up over the years. It’s that capital that we’ve invested in each other which I think is allowing our plans now to take shape.” (P16_IP01)*

#### Communication between professionals

In order to facilitate continuous communication between professionals – and in some cases also the patients and/or their informal caregivers – many of the investigated programmes have implemented communication platforms like regular team meetings or case conferences.

In South Somerset Symphony [UK], there are regular team meetings in which the most complex cases are reviewed and discussed in a non-hierarchical and informal way, allowing everyone to bring up what they regard the most relevant issues. These so-called “huddles” are a key instrument for communication, as an initiator of the programme highlights:

*“[…] and that’s where they discuss all their patients who are ten on the Symphony scale so the ones they’re most worried about. They tend to be the ones who have just come into hospital, just come out from hospital, massive change in circumstances so if one of them has just gone into the hospice or something like that that’s changed for the patient, so they tend to be discussed on a daily basis. […] So the huddle is a key thing and tends to happen early in the day.” (P17_IP03)*

### Patient involvement

A central aspect that is intensively discussed in the literature on integrated care (e.g. [49–51]) and also surfaced in the analysis of the programmes is the involvement of patients in all stages of the care process. In contrast to a care approach in which the patient is a passive receiver of treatment, such efforts allow patients to actively contribute to their treatment options. In this context, we identified two main sub-themes, namely (1) joint goal-setting/shared decision-making and (2) adaptation to reorganised service delivery.

#### Joint goal-setting and shared decision-making between patients and professionals

Most of the investigated programmes put a special emphasis on involving the patient when setting goals for his/her treatment. This is of particular importance for persons with complex needs, as they often need to prioritise among possibly conflicting goals [52]. This is the case when it is too demanding or impossible to address multiple health and/or social problems simultaneously. Therefore, efforts to involve the patient in all decisions to be made in the care process are being increasingly propagated, especially in integrated care [53–56]. The opportunity to define goals and participate in the decision-making process is highly valued by many patients, as several interviews across the programmes indicated. However, some patients leant towards entrusting the decisions to the professionals altogether. It seems that giving the choice to what degree patients are involved in decision making is beneficial to the care process.

In Gesundes Kinzigtal [DE], an individual treatment plan is developed together with the patient, following a goal-oriented approach. This treatment plan is based on realistic goals set by the patient, as this physician explains:

*“If I have a patient with, for example, overweight and diabetes, I try to actively involve him. I ask the patient: What can you contribute to the improvement of your health status? What are you willing to contribute? What is your aim for your personal health? Regardless whether the patient expresses the wish to be physically active, to reduce weight or to change the diet. Usually, I try to include the patient’s wish and adapt the treatment options accordingly in order to achieve the highest compliance and motivation. […] Treatment goals should always be feasible and achievable, hence adapted to the patient.” (P06_IP05)*

U-PROFIT [NL] aims at preserving physical functioning of frail elderly, so as to postpone or prevent admission to institutional care facilities. As a project manager points out, this aim must also be a priority for the patients themselves in order for the programme to be successful:

*“[Living at home longer is] what everyone essentially wants. That’s what the government really wants, but most older people too. And that only works if you link up with what someone finds important.” (P09_IP10)*

Learning Networks [NO] applies a tool for functional ability assessment called ‘What matters to you’, which combines the Patient-Specific Functional Scale (PSFS [57];) with priorities set by the patient. A nurse explains that this tool has been effective in improving patient involvement:

*“What matters to you’ has come into focus and been brought up much more with good patient pathways so I think user involvement has gotten through much more now than earlier, we maybe thought we had this before, but now we are much better at asking the user first.” (P13_IP14).*

Prioritising a patient’s wishes is of particular significance in palliative care, as the manager of the Palliative Care System [HR] stresses:

*“The person's wishes, their needs, their pain, and their torments are more important than the treatment protocols. It is more important to respect that than to get the result that using some medicine can prolong the patient's life for 7 or 15 days which can be published in conferences. We really care about the person.” (P04_IP03)*

#### Adaptation to reorganised service delivery

A prerequisite for being able to actively involve the patient in the care process is that he/she accepts and adapts to the reorganisations in service delivery that the programme entails. This aspect has emerged in the context of several programmes. While the favourable effects of patient involvement were acknowledged in most programmes, some interviews indicated that such measures can also be demanding and challenging for both persons with complex needs and professionals.

In South Somerset Symphony [UK], for example, a physician acknowledges that a shift in patients’ mind-set is necessary in order to accept the new care model, in particular the physician not being the patient’s primary contact point anymore:

*“I mean some patients love it, some patients, you know, are used to their GP and, you know, does my GP not want me anymore… The doctor is not always the first point of contact as it happens in primary care, because that is just unsustainable, we all have to – everybody has to change... Patients have had to get used to the fact that they may not see a doctor as much as they did when they were […] able to access them through primary care, which is often a reason why they are referred to us because, you know, the demand on primary care is so great.” (P17_IP06)*

In some programmes, informal caregivers play a central role in the delivery of care and are therefore also required to adapt to new situations. This is, for example, the case in Badalona Serveis Assistencials [ES], which is focused around home hospitalisation. As the quote from a physician below illustrates, changes entailed by the intervention can be challenging for informal caregivers. The programme therefore follows the strategy to simplify and dose the guidelines according to informal caregivers’ capabilities and, if necessary, schedule more frequent visits:

*“Some caregivers on the one hand have difficulties to understand the intervention, and on the other hand there are resistances to change habits. They are used to do things in one way and when you say ‘now you’ll mobilise him [the patient] this way’ it is difficult for them to understand the cure plan. It’s not that they are not willing but that they have difficulties to understand. Especially during the first days of the intervention, when they get much information.” (P14_IP05)*

## Discussion

The existing literature on integrated care provides both theoretical frameworks to describe what aspects are required for integration of care (e.g. [22, 58–61]) and a multitude of single case studies on programmes, many of which target single diseases only. Our article adds to the existing literature in various respects. The results are derived from an analysis of 17 programmes in eight European countries, covering a broad spectrum of integrated care approaches for persons with complex needs. Conducting a large number of interviews – 233 in total – enabled us to include perspectives of various different stakeholders. While adopting such a comprehensive scope, we applied a uniform analytical approach that allowed for an open and profound investigation of the actual processes of care and interpersonal relationships that shape these programmes in practice. Whereas some of the facilitators of a well-functioning care process have previously been posited in the literature, in our analysis, they emerged from numerous individual stakeholder perspectives. Furthermore, our analysis highlights the specific importance of these aspects in the care of persons with complex needs as it focuses on care approaches that explicitly target this population group.

A holistic view of the person and his/her needs and environment emerged to be a commonly employed success factor in care delivery to persons with complex needs. Even when the focus lies on the treatment of physical conditions, in many of the programmes, mental health and the social situation are routinely addressed to facilitate treatment of physical health problems. While the interconnectedness of physical health, mental health and social circumstances has been acknowledged in the literature [36–40], our analysis shows how much this connection matters on a practical level to several stakeholder groups.

Coordination of care is generally seen as an important element for integrated care [58, 60, 62, 63]. As cases become more complex, coordination and continuity become more important, but also more challenging, as it requires professionals to take on new roles and tasks. However, we have found that coordination efforts are valued by patients in our real-world examples, as they improve experience of care. Several of the investigated programmes created the function of a single contact point for patients and/or their informal caregivers who provides support in navigation through often fragmented health and social care systems. In accordance with the patient’s main problem area, this role is assumed by carers with very diverse professional backgrounds. Furthermore, multi-disciplinary teams are a key feature of integrated care [43–45, 54], which enables patients to receive a wide range of services that are still aligned and from one common source. Considering the various different professional backgrounds coming together in these teams, good communication, but also trust that can only be built over time emerged as highly important aspects. In order to achieve this, programmes provide platforms for continuous eye-level communication among all involved professionals (e.g. regular team meetings, case conferences). The requirement of building trusting relationships over time should also be held in mind when attempting to transfer care models, as implementing collaborative structures in a top-down fashion may not be successful.

While there are typically several professionals involved in the care of persons with complex needs, the person that should be at the centre of the care process is the patient him−/herself. The investigated programmes put the concept of goal-oriented medicine [52, 64, 65] into practice by giving the patient the opportunity to set priorities and take part in decisions throughout the care process. Some programmes also involve informal caregivers in the planning of care, as their role tends to become more important with increasing complexity. However, it has also emerged from our analysis that patients and informal caregivers need to be given the chance to adapt to changes in service delivery and that both the scope and the type of involvement have to be tailored to their individual abilities. Hence, support measures should be designed so that the patient is neither patronised nor left alone.

Although we base our analysis on 233 interviews from 17 programmes, owing to the methodological approach rooted in the qualitative paradigm, there are natural limitations. While all programmes address complexity of treatment in an integrated care framework, we do acknowledge the various differences between the programmes regarding their aims, target populations as well as the cultural and institutional context they are set in. We addressed elements of care that are present in most or all of the programmes and vital for their successful real-life implementation, although these are shaped according to the specific programme. One has to be aware that our insights are based on a limited number of personal views and experiences and there cannot be a one-size-fits-all model of integrated care for persons with complex needs. However, we believe that the aspects we identified in our analysis can serve as guidance when designing such programmes and creating adequate framework conditions for their successful functioning.

## Conclusions

Based on a highly explorative research approach, we were able to identify several key aspects for a well-functioning integrated care process for complex patients and how these are put into actual practice. The following four are discussed in the article: holistic view of the patient, continuity of care, relationships between professionals, and patient involvement. We investigated, on the one hand, how patients, informal caregivers and professionals view these aspects, and, on the other hand, how they are incorporated into the care process by the various programmes.

The identified aspects can be regarded as important in all chronic care, but are of particular significance and associated with increased challenges in the context of care of persons with complex needs. Thus, it is essential to incorporate these in the design of care approaches. However, our analysis has also highlighted that such care models cannot be planned on the drawing board. The development of mutual trust and commitment among all involved stakeholders seems paramount for the reorganisation of services and adaptation to new practices. Policy makers are therefore called to create the appropriate conditions to enable better design, implementation and spread of successful programmes taking these findings into account.

The field of integrated care for persons with complex needs leaves considerable room for further research. Topics worth investigating in this context include integrated care workforce (e.g. development of new professional roles), sustainable financing and payment schemes, efficient governance structures and potential for digital solutions in integrated care.

## Supplementary Information

**Additional file 1.**

## Data Availability

The 17 thick description reports are available under www.selfie2020.eu/publications. Audio recordings and transcript data of individual interviews are not available for third persons as this would breach anonymity, which was assured to interviewees.

## Abbreviations

Ais-Be: Área Integral de Salut, Barcelona Esquerra
AT: Austria
BSiN: Better Together in Amsterdam North
DE: Germany
ES: Spain
EU: European Union
GP: General practitioner
HR: Croatia
HU: Hungary
NL: Netherlands
NO: Norway
SELFIE: Sustainable intEgrated chroniccare modeLs for multi-morbidity: delivery, FInancing, and performance
UK: United Kingdom
U-PROFIT: Proactive Primary Care Approach for Frail Elderly
